# Synopsis of the Report of Analyses of Ohio River Water

**Published:** 1881-03

**Authors:** E. G. Betty


### SYNOPSIS OF THE REPORT OF ANALYSES OF OHIO RIVER WATER.

BY E. G. BETTY, D. D. S.

As the majority of the *Register's* readers do not reside in Cincinnati, they consequently do not partake of its waters in their ordinary form. It may, nevertheless be of interest to them to learn something about the "element" that enters so largely into the manufacture of our beer, for who has not heard of, and possibly imbibed that seductive beverage.

Its reputation is world wide, and it is not improbable but that it has made itself known in all the byways and X roads, Confederate and otherwise. At all events, we can all try and taste the sewage in it when the Miss. Val. Society in session.

A story is told about a King of India who dashed to pieces a microscope because it revealed to his astonished gaze the animalcule in his drinking water. We hope no one reading this article will send the chemist anonymous letters adorned with artistic skull and cross-bones, threatening him with vengeance dark, dire and dismal.

Joking aside, we have heretofore not been well informed as to the chemical characteristics of the water we consume. The recent analyses by Prof. C. R. Stuntz, of Woodward High School, supply a serious and long felt want. They were made under the auspices of the Board of Health, and independently of their official origin are of value as coming from an analyst of high repute.

"The water supply of Cincinnati comes from four sources, each of which furnishes a kind of water differing in properties and general character from all the rest."

First, and most important, the Ohio river.

Second, spring water, having its origin in rains and snows falling upon the higher land and percolating through the soil to lower levels.

Third, deep well water; a well deeper than twelve hundred feet is a true artesian, all characterized by the presence of sulphur.

Fourth, underground cisterns for catching rain water.

The Professor has analized all these waters, but it is with the first we are more nearly concerned.

"1. A comparison of the water drawn from the river at the pumping works with that in Eden Reservoir, with the water at Markley Farm, and also with that of the river below the general outflow of the sewage of the city.

2. A comparison of water filtered through the sand of the river beach near Dayton, Kentucky, with the other waters under examination.

3. The general condition of the Ohio River water in comparison with the water supply of other cities.

The basis of comparison of drinking waters is the amount of foreign organic and mineral ingredients considered especially with reference to the presence of sewage."

These extracts will serve to elucidate the accompanying tables, not forgetting that "the amount of ammonia and chlorine indicate the comparative amount of sewage present."

"The free ammonia indicates the comparative amount of nitrogenous organic matter, that has recently been subjected to putrifactive decomposition, and the so-called albumenoid ammonia the comparative amount of nitrogenous organic matter waiting in the water for the action of decomposing agents."

Water containing less .0100 of ammonia is superior drinking water; .0100 to .0150 good; .0150 to .0200 not good; .0200 to .0250 bad; more than .0250 very bad.

"The numerical expression of the comparative amount of sewage may also be made by the method suggested by Wanklyn, which consists in multiplying the so-called albumenoid ammonia by ten (10) for the entire sewage. In the following tables this amount is computed in aveirdupois pounds to the million United States gallons."

"Waters exposed to atmospheric air contain natnrally about one pound to one and one-half of sewage to the million gallons.

On this basis we have the general conditions of comparison:

| | | |
|---|---|---|
| Croton water, New York City......... | .98 | lbs. sewage to the 1,000,000 gal. |
| Loch Katrine, Glascow................. | .66 | " " " " |
| Thames, London supply............... | .50 | " " " " |
| Mystic River, Boston, Mass............ | 1.83 | " " " " |
| Fresh Pond, Cambridge, Mass......... | 1.50 | " " " " |
| Fairmount, Philadelphia................ | 1.58 | " " " " |

*1st. General Condition of Ohio River Water.*

| | | |
|---|---|---|
| Dayton Sand Beach (filt'd water)... | .82 | lbs. sewage to the 1,000,000 gal. |
| Markley Farm.............................. | 1.90 | " " " " |
| Pumping Works........................... | 1.81 | " " " " |
| Eden Reservoir............................ | 1.78 | " " " " |
| Storrs........................................ | 1.96 | " " " " |
| Eggleston Avenue sewer................. | 4.41 | " " " " |

*2d. Worst Condition.*

| | | |
|---|---|---|
| Dayton Sand Beach........................ | 2.03 | lbs. sewage to the 1,000,000 gal. |
| Markley Farm.............................. | 5.23 | " " " " |
| Pumping Works........................... | 11.39 | " " " " |
| Eden Reservoir............................ | 6.07 | " " " " |
| Storrs........................................ | 10.00 | " " " " |
| Eggleston Avenue sewer................. | 17.91 | " " " " |

*3d. General Average from Analysis.*

| | | |
|---|---|---|
| Dayton Sand Beach........................ | 1.35 | lbs. sewage to the 1,000,000 gal. |
| Markley Farm.............................. | 2.17 | " " " " |

| | | | | | |
|---|---|---|---|---|---|
| Pumping Works | 4.18 lbs. sewage to the 1,000,000 gal. | | | | |
| Eden Reservoir | 3.17 | " | " | " | " |
| Storrs and Lower River | 5.94 | " | " | " | " |
| Eggleston Avenue sewer | 11.16 | " | " | " | " |

The above tabulated results are deduced from elaborate tables the chemist has prepared, and which are embodied in his report. They are sufficient for our purpose. A supplementary report on cistern water is also made by Prof. Stuntz, from which the Health Board Committee draw a few rules to be observed by all who depend upon rain water in cisterns:

"1. Cisterns should be repeatedly and thoroughly cleansed, and especially those receiving water from roofs of dwellings.

"2. The drainage away from the cistern should be perfect.

"3. The cistern should be located as far from the privy vault as possible, and care should be taken that the matter in the privy vaults be kept constantly below the level of the bottom of the cistern.

"4. Cistern water should be drawn by means of buckets, chain pumps, or such other means as will introduce plenty of fresh air into the water."

